# *In Vivo* Determination of Vitamin D Function Using Transgenic Mice Carrying a Human Osteocalcin Luciferase Reporter Gene

**DOI:** 10.1155/2013/895706

**Published:** 2013-03-27

**Authors:** Tomoko Nakanishi, Rumiko Saito, Makoto Taniguchi, Haruka Oda, Atsumi Soma, Mayu Yasunaga, Mariko Yamane, Kenzo Sato

**Affiliations:** ^1^Division of Molecular Biology, School of Life Sciences, Faculty of Medicine, Tottori University, Yonago, Nishicho 86, Tottori 683-8503, Japan; ^2^Chromosome Engineering Research Center, Tottori University, Yonago, Tottori 683-8503, Japan

## Abstract

Vitamin D is an essential factor for ossification, and its deficiency causes rickets. Osteocalcin, which is a noncollagenous protein found in bone matrix and involved in mineralization and calcium ion homeostasis, is one of the major bone morphogenetic markers and is used in the evaluation of osteoblast maturation and osteogenic activation. We established transgenic mouse line expressing luciferase under the control of a 10-kb osteocalcin enhancer/promoter sequence. Using these transgenic mice, we evaluated the active forms of vitamins D2 and D3 for their bone morphogenetic function by *in vivo* bioluminescence. As the result, strong activity for ossification was observed with 1**α**,25-hydroxyvitamin D3. Our mouse system can offer a feasible detection method for assessment of osteogenic activity in the development of functional foods and medicines by noninvasive screening.

## 1. Introduction

Vitamin D is an essential factor for ossification, including activation of calcium absorption in the intestine, inhibition of calcium release in the kidney, and promotion of osteogenesis in bone. Indeed, vitamin D deficiency is well known to induce bone-softening diseases, such as rickets in children and osteomalacia in adults [1, 2]. Vitamin D is generated as previtamin D from 7-dehydrocholesterol by ultraviolet light irradiation of the mammalian skin and absorbed from various foods [3]. Animal foods contain vitamin D3 (cholecalciferol), while vegetable foods are enriched in vitamin D2 (ergocalciferol). The active form of vitamin D, 1*α*,25-hydroxyvitamin D [1*α*,25(OH)_2_D], is converted from previtamin D in the liver followed by the kidney [4, 5]. 

Osteocalcin, which is a noncollagenous protein found in bone matrix and involved in mineralization and calcium ion homeostasis, is one of the major bone morphogenetic markers and is used in the evaluation of osteoblast maturation and osteogenic activation [6–8]. The osteocalcin gene is regulated by various growth factors, hormones, cytokines, and vitamins. Basic fibroblast growth factor [9], bone morphogenetic proteins 2 and 4 [10, 11], and parathyroid hormone [12] are the major positive regulatory factors for osteocalcin gene expression, as well as vitamin D. The promoter region of the osteocalcin gene contains some transcriptional regulatory elements, such as the AP-1/VDRE (AV) element composed of a vitamin D-responsive element (VDRE), retinoic acid-responsive element (RE), and Jun-Fos-responsive AP-1 [13], as well as osteoblast-specific factor-binding elements (OSE1 and OSE2) [14]. The vitamin D receptor activated by association of vitamin D promotes transcription of the osteocalcin gene through interaction with a VDRE in the promoter of the gene [15].

Previously, we produced a transgenic mouse line expressing luciferase under the control of a 10-kb human osteocalcin enhancer/promoter sequence. This mouse line was backcrossed with a hairless mouse line to enable us to monitor bone formation during growth, fracture repair, and aging using *in vivo* imaging, without sacrificing the mice [16]. Using this system, we evaluated vitamin D function using osteocalcin gene expression as an indicator. 

## 2. Materials and Methods

### 2.1. Ethics Statement

All of the animal experiments described were approved by the Institutional Animal Care and Use Committee of Tottori University (Permission nos. 18-2-42 and 09-Y-64). All the mice received humane care in compliance with Tottori University's guidelines for the care and use of laboratory animals in research.

### 2.2. Cell Culture and Reporter Assays

MG-63 human osteosarcoma cells and HeLa cells were cultured in Eagle's minimal essential medium and Dulbecco's modified Eagle's medium supplemented with 10% fetal bovine serum (Thermo Fisher Scientific Inc., Waltham, MA), 100 U/mL penicillin (Meiji Seika Pharma Co. Ltd., Tokyo, Japan), and 0.1 mg/mL streptomycin (Meiji Seika Pharma Co. Ltd.) at 37°C under 5% CO_2_ in air. For luciferase reporter assays, 2 *μ*g of pOC-Luc (luciferase gene with the human osteocalcin enhancer/promoter) or pΔOC-Luc (luciferase gene without the osteocalcin enhancer/promoter) was cotransfected into MG-63 or HeLa cells (1 × 10^5^) using TransIT-LT1 (Mirus Bio LLC, Madison, WI) with 0.1 *μ*g of pRL-TK (TOYO B-Net, Tokyo, Japan), a plasmid carrying the *Renilla* luciferase gene driven by the thymidine kinase minimal promoter as an internal control. Dimethylsulfoxide (DMSO) as the vehicle or 1 nM of previtamin D2 (Sigma-Aldrich Co., St. Louis, MO, USA), previtamin D3 (Sigma-Aldrich), 1*α*,25(OH)_2_D2 (Sigma-Aldrich), and 1 nM 1*α*,25(OH)_2_D3 (Merck KGaA, Darmstadt, Germany) in DMSO (0.01% final DMSO concentration) were added at the same time as the transfection. At 24 h after the transfection, the cells were solubilized and the luciferase activities were measured using a Pikkagene Dual Luciferase Assay System (TOYO B-Net). The firefly luciferase activity was normalized by the *Renilla* luciferase activity in the same sample. 

### 2.3. *In Vivo* Luminescence Imaging

Hairless human osteocalcin enhancer/promoter-luciferase transgenic mice (OC-Luc Tg mice) [16] were anesthetized with isoflurane (DS Pharma Animal Health Co. Ltd., Osaka, Japan) at 8–11 months of age and then injected subcutaneously with luciferin (Promega, Madison, WI) at a dose of 160 mg/kg body weight (40 mg/mL luciferin). After 8 min, the mice were placed on their ventral surface, and images of the luciferase activity were continuously acquired every 3 min until the maximal activity was detected using an IVIS Lumina Imaging System (Xenogen Corp., Alameda, CA). The luciferase activity tended to reach its peak at around 15 min. To analyze the responsiveness to previtamin D3 (Sigma-Aldrich Co.), previtamin D2 (Sigma-Aldrich Co.), 1*α*,25(OH)_2_D3 (Merck KGaA), and 1*α*,25(OH)_2_D2 (Sigma-Aldrich Co.), the luciferase activity was measured at 0, 6, 9, and 24 h after oral administration of each agent at a dose of 2 ng/g body weight. The acquired images were analyzed using Live Image 2.6 software (Xenogen Corp.) to quantify the luciferase activity.

### 2.4. Statistical Analysis

Statistical analysis was performed using StatView (SAS Institute Inc., Cary, NC). The Student's *t*-test was used to analyze the difference between the study and control groups.

## 3. Results and Discussion

### 3.1. Response of the Human Osteocalcin Enhancer/Promoter-Luciferase Construct to 1*α*,25(OH)_2_D3 *In Vitro*

**Figure 1:**
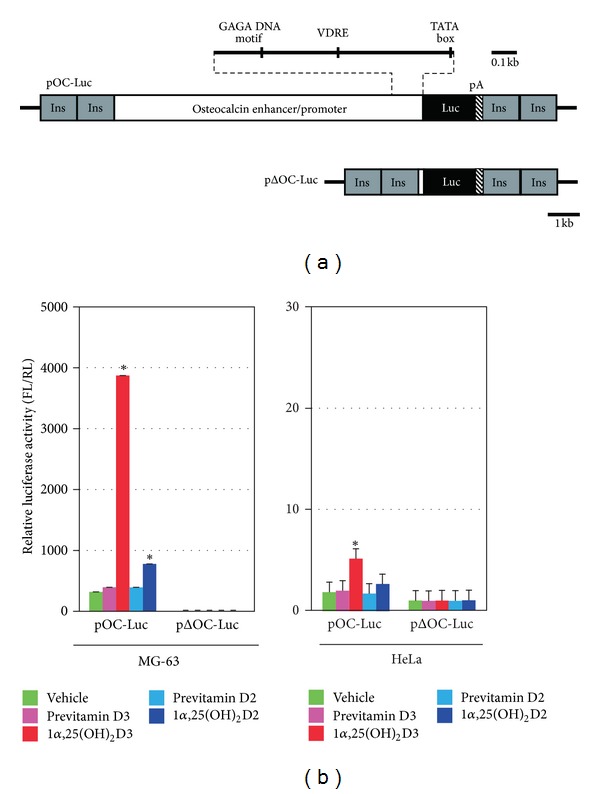
Constructs for the generation of the transgenic mice. (a) Schematic map of the transgenes used in the study. pOC-Luc is composed of a 10-kb human osteocalcin enhancer/promoter sequence, with 60 bp of the 5′-untranslated sequence (white box), a luciferase gene (black box), an SV40 late polyadenylation signal (striped box), and an insulator sequence (gray box). (b) Enhanced expression of pOC-Luc by 1*α*,25(OH)_2_D3 treatment. MG-63 and HeLa cells transfected with pOC-Luc or pΔOC-Luc were incubated with 1 nM previtamin D3, 1*α*,25(OH)_2_D3, previtamin D2, 1*α*,25(OH)_2_D2, or vehicle, and then subjected to luciferase reporter assays. The *y*-axis shows the relative luciferase activity, representing the firefly luciferase (FL) activity from the reporter plasmid normalized by the *Renilla* luciferase (RL) activity from the control vector. Statistical analysis was performed by Student's *t*-test; *P < 0.005 relative to vehicle.

We previously constructed a plasmid expressing luciferase under the control of a 10-kb human osteocalcin enhancer/promoter sequence (pOC-Luc) (Figure 1(a)) [16]. Osteocalcin is a gene that is predominantly expressed in bone-associated tissues. The fragment contains a VDRE, a GAGA DNA motif, which is suggested to control the 1*α*,25(OH)_2_D3 responsiveness of the rat osteocalcin gene [17, 18], as well as a TATA box. The osteocalcin translation start codon, ATG, was fused to the ATG of the luciferase gene. To insulate the transgenes from chromosomal position effects in the transgenic mice, two copies of the 1.2-kb chicken *β*-globin 5′-HS4 element were inserted at both ends.

The regulation of pOC-Luc was monitored by its luciferase activity following transfection into MG-63 cells. The MG-63 cell line is a well-characterized human osteoblast-like cell line that shows 1*α*,25(OH)_2_D3-dependent stimulation of osteocalcin production [16, 19]. The luciferase activity of pOC-Luc was high compared with that of pOCΔ-Luc, containing approximately 60 bp of the 5′-untranslated sequence. When the transfected cells were treated with 1*α*,25(OH)_2_D3 and 1*α*,25(OH)_2_D2, the luciferase activity of pOC-Luc was stimulated by approximately 10- and 2-fold, respectively (Figure 1(b)). On the other hand, there was no stimulation by previtamin D3 and D2 treatment. In HeLa cells, the pOC-Luc activity and the induction by 1*α*,25(OH)_2_D3 and 1*α*,25(OH)_2_D2 was less compared to MG-63 cells (Figure 1(b)). These findings suggest that pOC-Luc would be a useful tool for measuring bone-specific 1*α*,25(OH)_2_D3 induction of the human osteocalcin gene.

### 3.2. Effects of Oral Administration of Vitamins D3 and D2 on Osteocalcin Gene Expression in OC-Luc Tg Mice

We previously established a transgenic mouse line, OC-Luc Tg, harboring the human osteocalcin enhancer/promoter-luciferase gene derived from pOC-Luc [16]. When the OC-Luc Tg mouse line was backcrossed to a hairless mouse line, bioluminescence was observed along the bones after luciferin administration by *in vivo* imaging [16]. Consistent with the response of MG-63 cells to 1*α*,25(OH)_2_D3, the bioluminescence of the whole body of OC-Luc Tg mice after intraperitoneal injection of 1*α*,25(OH)_2_D3 was more than twice that measured in mice treated with vehicle [16]. 

**Figure 2:**
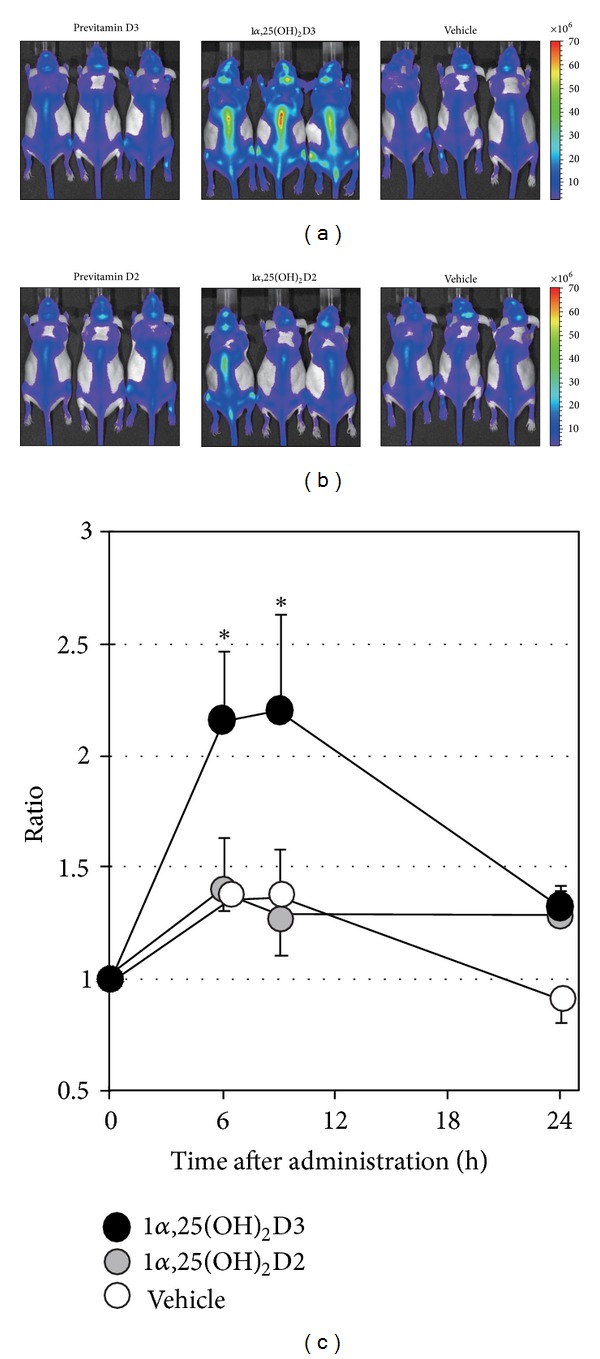
Regulation of the human osteocalcin enhancer/promoter by vitamins D3 and D2 *in vivo*. (a) Enhanced expression of the OC-Luc transgene by 1*α*,25(OH)_2_D3 treatment. OC-Luc Tg mice were given a single oral administration of previtamin D3, 1*α*,25(OH)_2_D3, or vehicle. *In vivo* bioluminescence imaging was performed at 6 h after the administration. (b) Response of the OC-Luc transgene to 1*α*,25(OH)_2_D2 treatment. OC-Luc Tg mice were given a single oral administration of previtamin D2, 1*α*,25(OH)_2_D2, or vehicle. *In vivo* bioluminescence imaging was performed at 6 h after the administration. (c) Time course of the induction of the OC-Luc transgene by vitamin D treatment. OC-Luc Tg mice were given a single oral administration of 1*α*,25(OH)_2_D3 (filled circles), 1*α*,25(OH)_2_D2 (shaded circles), or vehicle (open circles). The bioluminescence was analyzed at 0, 6, 9, and 24 h after the administration using Living Image software and represented as the ratio of total flux (photons/second) compared to 0 h. The data shown are means ± SE (*n* = 3). Statistical analysis was performed by Student's *t*-test; *P < 0.05 relative to vehicle.

To compare the effects of 1*α*,25(OH)_2_D3, previtamin D3, 1*α*,25(OH)_2_D2, and previtamin D2 on the bone formation activity, we aimed to measure the alterations in human osteocalcin expression after oral administration of these agents to OC-Luc Tg mice. The bioluminescence of the whole body of OC-Luc Tg mice at 6 h after oral administration of 1*α*,25(OH)_2_D3 was more than twice those measured in mice treated with previtamin D3 or vehicle (Figure 2(a)). When the bioluminescence was measured at 6 h after oral administration of 1*α*,25(OH)_2_D2, weak induction was observed, similar to the case for mice treated with previtamin D2 or vehicle (Figure 2(a)). As shown in Figure 2(b), the increase in bioluminescence induced by 1*α*,25(OH)_2_D3 reached its maximum level at 6–9 h after oral administration and returned to the control levels by 24 h, as previously reported [16, 20], whereas weak induction was observed within 24 h after 1*α*,25(OH)_2_D2 administration. These findings indicate that only 1*α*,25(OH)_2_D3 is effective for increasing the bone formation activity after a single oral administration to OC-Luc Tg mice, compared with 1*α*,25(OH)_2_D2, previtamin D3, and previtamin D2. 

The bone-mobilizing activity of 1*α*,25(OH)_2_D2 was reported to be lower than expected from its vitamin D receptor (VDR) affinity, which is only 3 times less than that of 1*α*,25(OH)_2_D3 [21]. Differential metabolism is a potential mechanism for the analog selectivity. Therefore, it is possible that 1*α*,25(OH)_2_D2 may be catabolized more rapidly in bone. Another possible explanation for the difference in action between 1*α*,25(OH)_2_D3 and 1*α*,25(OH)_2_D2 is that 1*α*,25(OH)_2_D2 does not mimic 1*α*,25(OH)_2_D3 in upregulating the VDR. In addition, there is a lack of clarity in the literature as to whether there is a definitive difference between the effects of vitamins D2 and D3 on raising the serum 25-hydroxyvitamin D levels in humans [22]. The precise processes that increase the bone formation activity through vitamins D3 and D2 currently remain unclear. It may be important to evaluate the differences between vitamins D3 and D2 utilizing an indicator related to a specific biological process. 

## 4. Conclusion

In the transgenic mice harboring human osteocalcin enhancer/promoter luciferase reporter gene, strong osteogenic activity was observed by 1*α*,25(OH)_2_D3 administration, compared with 1*α*,25(OH)_2_D2, as well as previtamins D2 and D3. Our mouse system would offer a feasible detection method for assessing osteogenic activity in the development of functional foods and medicines by noninvasive screening.
